# Novice Shooters With Lower Pre-shooting Alpha Power Have Better Performance During Competition in a Virtual Reality Scenario

**DOI:** 10.3389/fpsyg.2018.00527

**Published:** 2018-04-12

**Authors:** Michael Pereira, Ferran Argelaguet, José del R. Millán, Anatole Lécuyer

**Affiliations:** ^1^École Polytechnique Fédérale de Lausanne, Defitech Chair in Brain-Machine Interface, Swiss Federal Institute of Lausanne, Geneva, Switzerland; ^2^Inria, Campus de Beaulieu, Rennes, France

**Keywords:** sports, attention, EEG, virtual reality, mu, alpha

## Abstract

Competition changes the environment for athletes. The difficulty of training for such stressful events can lead to the well-known effect of “choking” under pressure, which prevents athletes from performing at their best level. To study the effect of competition on the human brain, we recorded pilot electroencephalography (EEG) data while novice shooters were immersed in a realistic virtual environment representing a shooting range. We found a differential between-subject effect of competition on mu (8–12 Hz) oscillatory activity during aiming; compared to training, the more the subject was able to desynchronize his mu rhythm during competition, the better was his shooting performance. Because this differential effect could not be explained by differences in simple measures of the kinematics and muscular activity, nor by the effect of competition or shooting performance *per se*, we interpret our results as evidence that mu desynchronization has a positive effect on performance during competition.

## 1. Introduction

Experimental research in Virtual Reality (VR) has a lot of potential in different research fields such as experimental brain research, psychology (McClernon et al., 2011) and sports (Wellner et al., 2010). Among its advantages, VR minimizes disturbances in complex experiments involving multiple subjects or intricate experimental protocols. In particular, VR simulation enables the accurate control and synchronization of all the elements involved in the experiment and ensures reproducibility and comparison among the different trials. In addition, due to increased user immersion and presence in the virtual environment (Slater, 2009), the engagement and the motivation of the user can be ensured.

In sport training scenarios, which are the main focus of the study presented in this paper, the VR simulation allows to immerse athletes in controlled and realistic virtual environments reproducing a competitive scenario (for instance in this study: a shooting range, competitors, and an audience) enabling the study of behavioral and physiological entangled processes (Argelaguet et al., 2015). Competition can have various effects on athletes' performances. One such effect is “choking,” when athletes fail under pressure due to a stressing competitive environment. On the other hand, competition can have beneficial effects, such as a “clutch” performance, when athletes perform overly well under pressure (Ehrlenspiel, 2006).

Electrophysiological activity in the 10 Hz mu/alpha frequency range prior to pistol shots (Del Percio et al., 2009; Bertollo et al., 2016) or golf putts (Cooke et al., 2014) was shown to be related to performance and to dissociate experts from novices. However, conflicting evidence has been reported on the effects of competition on mu power, some studies finding reduced mu during competition (Hatfield et al., 2013) while others failing to find any effect (Cooke et al., 2014).

In this pilot study, we record novice shooters while using VR to induce a competitive shooting environment and explore both positive and negative effect of competition on the performance of novice shooters and the relation of these effects to mu power. While novice shooters are not the ultimate target population, they are arguably more subject to mild choking during controlled experiments than trained athletes.

## 2. Methods

### 2.1. Subjects

Nine healthy subjects (age: 25.6 ± 0.87 mean ± standard error of the mean) volunteered to participate in this study. Subjects reported no neurological or psychiatric problems and had normal or corrected-to-normal vision. The study was designed in accordance to the declaration of Helsinki, data was anonymized and all subjects provided prior written informed consent. Due to the minimal risk for subjects, at the time of data collection, no explicit approval form an ethical committee was sought. All subjects were right-handed. Data from one subject had to be discarded due to technical problems.

### 2.2. Virtual reality environment

**Figure 1:**
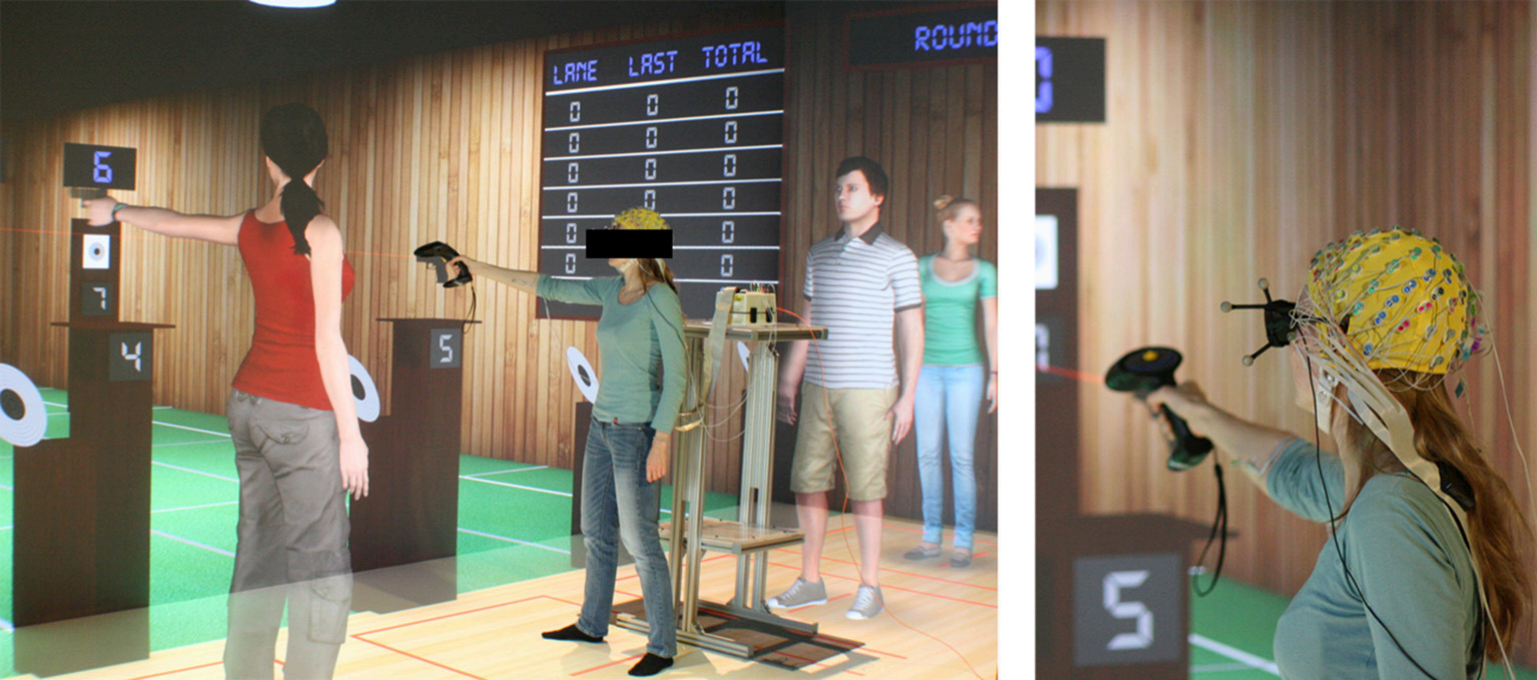
Experimental Setup. **(Left)** Subjects were standing in the immersive projection system and were able to interact with the system using an ART Flystick. **(Right)** Subjects were wearing a high-density 64 channels EEG cap.

The experiment was run in a four-sided immersive projection room with retro-projected glass screens. The dimensions of the immersive room were 9.6 × 3.1 × 3.1 m located at the Inria/IRISA laboratory at Rennes, France. The subject, head, hand position and orientation were tracked by 16 ART Tracking infrared cameras. The entire system was driven by a cluster of 14 workstations. The virtual environment reproduced a 10 m Olympic shooting environment in which participants had to aim and shoot at a 17 × 17 cm target from a 10 m distance. The virtual range had seven lanes which allowed to add six virtual characters. The virtual shooting range was 9.6 m wide which matched the size of the CAVE (see Figure 1). Additional details regarding the virtual shooting range, the avatar behavior and behavior of participant can be found in Argelaguet et al. (2015).

### 2.3. Experimental protocol

After reading and signing the consent form, subjects performed 2 min of eyes-opened resting state recording (baseline).

The experimental task consisted of aiming and shooting at a 17 × 17 cm target (0–10 points) placed at 10 m. Each aiming period started when the subject raised the gun with an aiming accuracy of < 2°. Then the subjects were instructed to aim at least 5 s before making a shot by gently pulling the trigger. Aiming was facilitated by displaying a virtual laser pointer and instructions were provided to ensure that all participants followed the exact same protocol.

Accuracy feedback (0–10) was provided to the subjects after each shot based on the distance between the shot and the center of the target.

Participants performed blocks of 10 shots. In each block, subjects were either alone in the shooting range (*training condition*), or competing against six virtual avatars with unsupportive crowd noises (*competition condition*).

Participants had to perform 80 shots in total, with a pause in the middle in order to rest their arm until they were ready to continue. Half of the subjects did two training blocks, followed by four competition blocks and finally two training blocks. The other half performed the experiment in the reversed order. The experiment lasted approximately 30 min in total.

### 2.4. Electrophysiological recordings

We recorded high-density electroencephalography (EEG) at 2,048 Hz in an extended 10–20 system with a Biosemi ActiveTwo amplifier. Data were downsampled to 512 Hz using the OpenViBE framework (Renard et al., 2010). The signals were then re-referenced (offline) to a common average. Electrooculographic (EOG) activity was measured using three additional sensors placed above the nasion and below the outer canthi of the eyes. Horizontal EOG was defined as the difference between the left and right outer canthi signals while vertical EOG was defined as the difference between the nasion and the mean of the two outer canthi signals. EOG signals were highpass filtered (zero-phase Butterworth filter, cutoff: 1 Hz). Finally, bipolar electromyographic (EMG) activity was recorded over the *flexor digitorum*, highpass filtered (Butterworth, cutoff: 60 Hz) and the envelope was computed using a Hilbert transform.

Aiming epochs were defined as the 5 s of data preceding a trigger pull. The 2 min eyes open baseline constituted the resting state data. For each aiming epoch and the resting state data, we computed the Welch spectrum by averaging the power of fast Fourier transform (FFT) spectra from 50% overlapping 500 ms windows. The mean of each window was subtracted prior to applying the FFT to remove slow drifts of the EEG signal. Windows were eliminated from the average if the maximum amplitude difference of either the horizontal or vertical EOG was higher than 70 μ*V*. Epochs with more than 50% of EOG-contaminated windows were discarded from further analysis.

## 3. Results

On a range from 1 to 7 (1 not at all, 7 a lot), subjects reported being competitive (5.94 ± 0.33), having medium VR experience (4.19 ± 0.68) and little shooting experience (2.38 ± 0.53). Participants rated whether the sound of the public was annoying (2.94 ± 0.66) and whether their performance was influenced by scores of avatars (4.19 ± 0.46). Mean shooting scores did not differ (non-parametric sign test, *p* = 0.73) between training (7.02 ± 0.25) and competition (7.14 ± 0.26).

**Figure 2:**
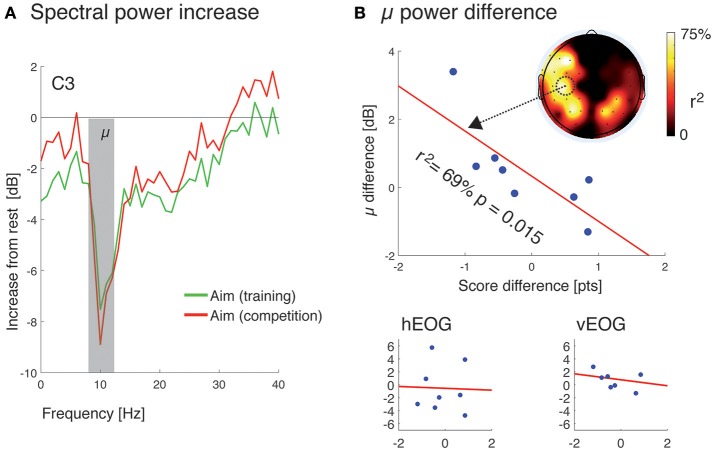
Experimental results: **(A)** Desynchronization of sensorimotor rhythm during shooting compared to resting was maximum in the mu (8–12 Hz) frequency band. **(B)** Differential effect of competition on score and mu power correlation over electrode C3. The topographic scalp distribution of the correlation is shown on the upper right corner. Repetition of the procedure using horizontal (bottom-left) and vertical (bottom-right) EOG showed no significant correlation.

For the electrophysiological analysis, we chose (a-priori) to focus our analysis on the C3 electrode as it overlies the hand representation of the sensorimotor cortex. The task-related desynchronization was indexed as the decrease in cortical oscillatory power before the trigger pull (in dB). Compared to resting state data, this desynchronization was maximal at 10 Hz (Figure 2A).

To compare mu power between the training and the competition conditions, we averaged frequencies between 8 and 12 Hz (mu band). No statistically significant difference was found (non-parametric sign test, *p* = 0.73) between training (−5.39 ± 1.50 [dB]) and competition (−2.07 ± 1.34 [dB]). Next, we sought to find a possible differential effect, i.e., whether the effect of competition on the electrophysiology would correlate with the effect of competition on behavioral performance (score). For this, we regressed the mu difference (competition-training) from the score difference (competition-training). A significant (*p* = 0.015) portion (69.4%) of the variance of the mu differences could be explained by the differences in score. This fitting was highest for the C3 electrode and more generally over the left sensorimotor area as well as the left parieto-occipital region (Figure 2B). Additionally, a control analysis performed on the horizontal and vertical EOG signals did not show any significant fitting (*r*^2^ = 0.2 and 0.9%, *p* = 0.93 and 0.84, respectively), precluding this effect from being due to ocular artifacts (Figure 2B).

To verify whether our results were not simply due to higher desynchronization during good shots, we also separated good (above median performance) from bad (below median performance) shots for every subject and found no differences between good and bad shots (non-parametric sign test, *p* = 0.29). Since the mu rhythm is a correlate of movement, we also verified whether muscular activity or kinematics differences could explain mu differences and repeated the analysis using EMG power differences (recorded over the *flexor digitorum*) and average speed of the arm (indexing lack of arm stability). Neither correlated significantly with mu differences (*r*^2^ = 8.1%, *p* = 0.50 and *r*^2^ = 16%, *p* = 0.33, respectively).

## 4. Discussion

We investigated effects of competition in shooting on mu frequency over the motor cortex. We used virtual reality to immerse novice shooters in a realistic shooting environment. Subjects were either alone in the shooting range or competing against six virtual avatars with loud background noises from a (virtual) unsupportive audience.

Contrarily to previous reports, we did not find consistent differences in mu desynchronization for competition at the group level (Hatfield et al., 2013), nor did we find an effect for good versus bad shots (Del Percio et al., 2009), which could be due to the small sample size of our study. However, we found a differential effect on mu desynchronization; subjects whose performances were impeded by the competition condition compared to when shooting alone showed lower mu desynchronization during the aiming period preceding the trigger pull (compared to when aiming alone). On the contrary, subjects for whom the competition had a positive effect on shooting performances showed higher aim-related mu desynchronization. The scalp topography of this effect, peaking over the sensorimotor area contralateral to the shooting hand is consistent with the involvement of mu desynchronization in movement preparation and execution (Pfurtscheller and Aranibar, 1979; Pfurtscheller and Lopes, 1999; Cheyne, 2013). Interestingly, the effect was also significant over the bilateral parietal areas.

We interpret the results of this pilot study as evidence that increased mu desynchronization in the visuomotor loop can help subjects inhibiting task-unrelated stimuli competing for attention (see Klimesch et al., 2007) and have positive effect on performance. Importantly, the differential effect of competition found in this study could not be explained by differences in movement instabilities. It remains to show whether this effect can be generalized to expert shooters.

Our findings could be relevant in sports training to help athletes avoid choking under pressure during competition. Confirmation through further experimental validation is needed.

## Ethics statement

There was no explicit approval by an ethical committee for our study. However, please note that:

1. All subjects participating in the experiment gave written informed consent in accordance with the Declaration of Helsinki. In particular, the informed consent form described the experimental protocol, detailed the rights and obligations for the subjects, described any potential risks and asked explicitly their consent regarding the use of the data gathered during the experiment.
2. Due to the minimal risk in the experiment for the subjects (physically and mentally), the fact that data recording was anonymized, and that we were not dealing with sensible data, we considered that there was no need for an explicit approval of the ethical committee. Indeed, at the moment of the experiment (end of 2014), the institution where experiments took place (Computer Science background) didn't have the culture of collecting systematically an explicit approval of the ethical committee for this kind of experiments –this requirement has clearly changed over the last years.

## Author contributions

MP and FA contributed equally. AL and JM also contributed equally. All authors designed the experiment and wrote the paper. MP and FA collected data. FA developed the VR framework. MP analyzed the EEG data. JM and AL oversaw the project.

### Conflict of interest statement

The authors declare that the research was conducted in the absence of any commercial or financial relationships that could be construed as a potential conflict of interest.
